# Calcium signaling: an emerging player in plant antiviral defense

**DOI:** 10.1093/jxb/erad442

**Published:** 2023-11-06

**Authors:** Anna S Zvereva, Michael Klingenbrunner, Markus Teige

**Affiliations:** Department of Functional & Evolutionary Ecology, University of Vienna, Djerassiplatz 1, 1030 Vienna, Austria; Department of Functional & Evolutionary Ecology, University of Vienna, Djerassiplatz 1, 1030 Vienna, Austria; Department of Functional & Evolutionary Ecology, University of Vienna, Djerassiplatz 1, 1030 Vienna, Austria; University of Ghent, Belgium

**Keywords:** Calcium signaling, chloroplast, crop protection, organelles, plant defense, plant defense suppression, plant virus, viral targets

## Abstract

Calcium is a universal messenger in different kingdoms of living organisms and regulates most physiological processes, including defense against pathogens. The threat of viral infections in humans has become very clear in recent years, and this has triggered detailed research into all aspects of host–virus interactions, including the suppression of calcium signaling in infected cells. At the same time, however, the threat of plant viral infections is underestimated in society, and research in the field of calcium signaling during plant viral infections is scarce. Here we highlight an emerging role of calcium signaling for antiviral protection in plants, in parallel with the known evidence from studies of animal cells. Obtaining more knowledge in this domain might open up new perspectives for future crop protection and the improvement of food security.

## Teaser

Recent findings in plant virus research, coupled with comparisons to animal cells, indicate an important role of calcium signaling in plant antiviral responses with high potential for future investigation.

## Introduction

The spread of viral infections of crops has increased dramatically all over the world in recent years, causing substantial economic damage (Chauhan *et al.*, 2019; Jones, 2021). Aside from economic loss, these viral diseases pose a significant risk for human food security. The increasing speed of climate change is escalating this problem even further (Trebicki, 2020; Amari *et al.*, 2021). Due to increasing temperature, the virus-transmitting insects are able to expand their geographic distribution and their survival during overwintering, and as a consequence the number of generations of these vectors increases. This also alters the interaction with their natural enemy species (Skendzic *et al.*, 2021). On top of these factors, drought, excessive flooding, the increase of the concentrations of CO_2_ and ozone in the atmosphere, as well as UV-B levels, severely affects the host’s physiology and resistance against pathogens (Bastas, 2022). On the other hand, an emerging area of recent research is the mutualistic relationship between plants and viruses, as infected plants sometimes develop increased resistance to abiotic stress (Aguilar and Lozano-Duran, 2022; Gorovits *et al.*, 2022). Climate change may also indirectly affect the efficacy of insecticides. Moreover, adaptation to one environmental stress (e.g. insecticides) causes further increased thermotolerance in whiteflies, a vector for many plant viruses (Aregbesola *et al.*, 2019).

New ways of crop protection must be developed to cope with these inevitable developments. Disease control is mainly based on prophylactic measures to restrain viral spread, for example, using quarantine, certification, the destruction of infected plants, and the use of insecticides to control the virus-transmitting insects (Rubio *et al.*, 2020). The second antiviral protection strategy, which strongly capitalizes on the knowledge of plant–virus interactions, aims to produce genetically resistant varieties that can be exploited in agriculture (Rubio *et al.*, 2020). Therefore, elucidating the molecular mechanisms underlying plant–virus interactions during viral entry, replication, movement, and transmission might contribute to the development of new strategies for effectively combatting viral threats in agricultural settings.

To counteract viral infections, plants defend themselves by using several mechanisms to restrict viral replication and movement. The best-studied defense mechanisms are RNA-mediated gene silencing, innate immune receptor-based signaling, translational repression, ubiquitination- and autophagy-mediated protein degradation, and the resistance genes response. Typical hallmarks of the plant immune response are the generation of the hypersensitive response (HR) and the salicylic acid-induced systemic and acquired resistance (Calil and Fontes, 2017; Wu *et al.*, 2019). Viruses, in turn, have developed their own mechanisms to suppress different plant defense strategies. The primary strategy employed by all viruses to evade the host’s immune defenses is the high mutation rate of their genome during replication, which prevents the recognition of modified viral proteins by the host’s immune system. Moreover, certain viral proteins have the ability to selectively bind to components of various defense pathways. This leads to direct repression of these defenses or to the virus exploiting some of these defense pathways for their own benefit (Wu *et al.*, 2019), both of which ultimately advance viral replication and spreading.

Calcium (Ca^2+^) signaling plays a key role in setting up the innate immunity defense reactions downstream of both cell-surface and intracellular receptor proteins, which are activated by pathogen-associated molecular patterns and effector proteins, respectively (Aldon *et al.*, 2018). The defense reactions include signaling via mitogen-activated protein kinases (MAPKs), reactive oxygen species (ROS) production, salicylic acid (SA) accumulation, and extensive transcriptional reprogramming to induce the local and systemic immune response to various pathogens in plants (Garcia-Brugger *et al.*, 2006; Gilroy *et al.*, 2016; Choi *et al.*, 2017). In recent years it has become clear that functional Ca^2+^ channels are required for immunity against biotrophic and necrotrophic pathogens (Koster *et al.*, 2022; Xu *et al.*, 2022). These channels facilitate a rapid increase of the free Ca^2+^ concentration in the cytoplasm by releasing Ca^2+^ from the intra- and extracellular stores. This activates intracellular Ca^2+^ decoders, which subsequently activate their target proteins, leading to immune responses. Moreover, there is evidence that the immune signaling triggered by elicitor perception can, in turn, regulate the transcription of glutamate receptor-like (GLR) Ca^2+^ channels (Bjornson *et al.*, 2021). Another striking phenomenon is the formation of a resistosome, which consists of several nucleotide-binding leucine-rich repeat receptors (NLRs) that are activated by pathogen-derived effector proteins. The NLR oligomer forms a pore, which is inserted into the plasma membrane (PM) and allows the influx of Ca^2+^ as well as other cations from the apoplast into the cytoplasm. Such ion influx is essential for the HR, which restricts the spread of pathogens including viruses (Forderer and Kourelis, 2023; Ivanov *et al.*, 2023; Shepherd *et al.*, 2023).

The focus of this review is to summarize the emerging evidence that Ca^2+^ signals are essential for setting up most of the antiviral defense mechanisms and that alteration of Ca^2+^ signaling could be a universal strategy used by viruses to suppress plants’ defense responses. Remarkably, the mechanisms that viruses employ to hijack Ca^2+^ signaling of the host cell have been studied very well in animals, but they are clearly underexplored in plants. Since many fundamental molecular mechanisms are similar in all eukaryotes, it seems appropriate to expect that analogous mechanisms occur in plants, as the role of Ca^2+^ signaling is universal in both types of organism (Luan and Wang, 2021). Therefore, we will draw parallels throughout the review between animal and plant viral hosts in order to highlight the crucial aspects that need to be studied in plants in the future.

## Viruses manipulate the Ca^2+^ concentration in the cytosol and modify host intracellular Ca^2+^ sensor activity and viral protein stability

### Ca^2+^ pumps, channels, and fluxes

**Fig. 1.**
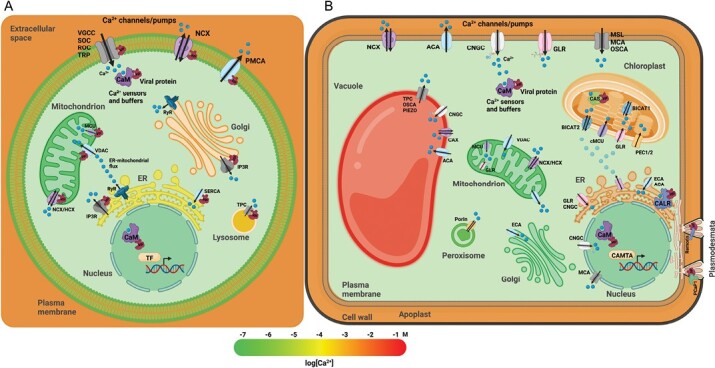
Ca^2+^ signaling perturbations caused by viral infection in animal and plant cells. (A) In animal cells, viral proteins interfere with the functions of Ca^2+^ pumps and/or channels at the plasma membrane (PM) and at the membranes of Ca^2+^ internal stores, including the endoplasmic reticulum (ER), Golgi apparatus, and mitochondria. Viral proteins interfere with Ca^2+^ release from internal stores accomplished by ryanodine receptors (RyR) and inositol-1,4,5-triphosphate receptors (IP3R). In animals and plants, viral proteins interact with intracellular Ca^2+^ sensors and buffers to remodel the Ca^2+^ signaling network or to activate or repress Ca^2+^-responsive transcription. (B) Plants have additional Ca^2+^ stores with the maximal concentration of Ca^2+^ in the cell: the central vacuole, the apoplast, and chloroplasts. In chloroplasts, a viral protein interacts with the chloroplast Ca^2+^ sensor (CAS) protein, suppressing CAS-dependent immune defense. The size-exclusion limit of the plasmodesmata regulates viral spread. Viral proteins regulate the size of plasmodesmata, facilitating cell-to-cell movement by competing for interaction with the Ca^2+^-binding protein pCaP1 and its interactor remorin. The gradient color bar at the bottom of the figure indicates the total Ca^2+^ concentration in the different subcellular compartments. Calcium ions are shown as blue dots. ACA, autoinhibited Ca^2+^-ATPase; BICAT 1, 2, bivalent cation transporter 1 and 2; CALR, calreticulin; CaM, Calmodulin; CAMTA, calmodulin-binding transcription activator; CAX, Ca^2+^-ATPases and Ca^2+^/H^+^ exchangers; cMCU, chloroplast-localized mitochondrial Ca^2+^ uniporter; CNGC, cyclic nucleotide-gated channel; ECA, ER-type Ca^2+^-ATPase; GLR, glutamate receptor-like channel; HCX, H^+^/Ca^2+^ exchanger; MCA, Mid1-complementing activity channel; MCU, mitochondrial Ca^2+^ uniporter; MSL, mechanosensitive-like channel; NCX, Na^+^/Ca^2+^ exchanger; OSCA, reduced hyperosmolarity-induced [Ca^2+^] cytosolic increase channel; PEC1/2, plastid envelope ion channel 1 and 2; PMCA, plasma membrane Ca^2+^-ATPase; ROC, receptor-operated channel; SERCA, sarcoplasmic/endoplasmic reticulum Ca^2+^-ATPase; SOC, store-operated channel; TF, transcription factor; TPC, two-pore channel; TR, transient receptor potential channel; VDAC, voltage-dependent anion channel; VGCC, voltage-gated Ca^2+^ channel. Figure created with Biorender.com. The template of the animal cell is redrawn from Zhou *et al.* (2009), with permission from Elsevier.

Viruses interfere with cellular Ca^2+^ signaling at many levels. In animals, regulation of the host antiviral response includes Ca^2+^-dependent activation of Toll-like receptors and dsRNA-sensing molecules, leading to the production of cytokines and interferons, the activation of immune cells, and inflammation (Qu *et al.*, 2022). In order to counteract host defenses, viruses apply different general strategies to manipulate regulators of Ca^2+^ signaling at intracellular levels to favor viral reproduction (Fig. 1A). First, binding to voltage-gated calcium channels (VGCCs) on the PM promotes fusion of the virion with the cell and virus entry (Qu *et al.*, 2022). Next, specific viral proteins are able to modulate the functions of various Ca^2+^ ion channels and pumps at the PM, such as the store-operated channel (SOC) and receptor-operated channel (ROC), the transient receptor potential channel (TRP), the PM Ca^2+^-ATPase (PMCA), and the Na^+^/Ca^2+^ exchanger (NCX), as well as the pumps and channels at the membranes of the Ca^2+^ internal stores. These include the sarcoplasmic/endoplasmic reticulum Ca^2+^-ATPase (SERCA), the mitochondrial Ca^2+^ uniporter (MCU), the H^+^/Ca^2+^ exchanger (HCX), and the voltage-dependent anion channel (VDAC) or the lysosomal two-pore channel (TPC). Additionally, in animals, Ca^2+^ ions can be released from internal stores through the inositol-1,4,5-triphosphate receptor (IP3R) and ryanodine receptor (RyR). As a result, a temporarily increased concentration of Ca^2+^ in the cytoplasm leads to activation of internal Ca^2+^ sensors such as calmodulins (CaMs) and protein kinases, which change the activity of their target proteins. This facilitates viral replication as well as suppression of the host’s defenses (Zhou *et al.*, 2009; Qu *et al.*, 2022).

Based on the observations of Ca^2+^-sensitive processes, it therefore seems likely that, similar to animal viral proteins, plant viral proteins would also directly target the channels and pumps that are responsible for the Ca^2+^ fluxes in and out of the cytoplasm (Fig. 1B). There are three plant-specific Ca^2+^ storage pools: the central vacuole, the extracellular space between the PM and the cell wall (the apoplast), and the chloroplast. Regulation of Ca^2+^ influx from the apoplast must be accomplished by PM-localized Ca^2+^ channels and pumps, such as GLRs, cyclic nucleotide-gated channels (CNGCs), mechanosensitive-like channels (MSLs), Mid1-complementing activity channels (MCAs), reduced hyperosmolarity-induced cytosolic [Ca^2+^] increase channels (OSCAs), autoinhibited Ca^2+^-ATPases (ACA, an analogue of animal PMCA) and NCXs (Luan and Wang, 2021). Some of these channels, being also present at the membranes of the different organelles, are responsible for the regulation of Ca^2+^ release from internal stores (Fig. 1B). Some Ca^2+^ channels and pumps are unique to specific plant organelles. The vacuolar membrane harbors the TPC, the Ca^2+^/H^+^ exchangers (CAXs), and PIEZO ion channels (Schonknecht, 2013). Mitochondrial membranes contain the MCUs and VDACs (Pirayesh *et al.*, 2021). In chloroplast membranes, the chloroplast-localized MCUs (cMCUs), the bivalent cation transporter 1 and 2 (BICAT 1, 2) (Frank *et al.*, 2019), and the POLLUX plastid envelope ion channel 1 and 2 (PEC1/2) have been reported (Volkner *et al.*, 2021). The endoplasmic reticulum (ER) membrane harbors the ER-type Ca^2+-^ATPases (ECAs), which are the analogs of animal SERCAs at the ER and presumably also at the Golgi apparatus (Edel *et al.*, 2017). Finally, the peroxisomal membrane also contains porins, which have been implicated in Ca^2+^ transport (Fig. 1B) (Pirayesh *et al.*, 2021).

It has been shown that the Arabidopsis putative ortholog of the animal PIEZO protein becomes activated upon viral infection and inhibits the systemic movement of the silencing suppressor-deficient cucumber mosaic virus (CMV) and the turnip mosaic virus (TuMV) (Zhang *et al.*, 2019). According to the authors of that study, the animal ortholog has a mechanosensitive cation channel activity. However, the cation transport function and the subcellular localization of this particular Arabidopsis PIEZO ortholog have not been determined. For the rest of the channels mentioned above, it is still unclear whether any of them contribute to the release of Ca^2+^ into the cytoplasm or to organelle-specific defense responses during viral infection. Thus, the question of whether they might be potential viral targets for the regulation of plant–virus interactions is still open, although available transcriptomics data would support this hypothesis. Chiu *et al.* (2021) identified by RNA-seq analysis that the transgenic expression in Arabidopsis of the TuMV protein P1/HcPro results in a significant change of genes that are responsible for the regulation of Ca^2+^ signaling at all levels. This included several calcium uniporters, calcium- and CaM-binding proteins, as well as several calcium-dependent protein kinases (CPKs). Then *et al.* (2021) demonstrated that several genes encoding Ca^2+^ channels such as GLRs and CNGCs were misregulated in cauliflower mosaic virus- and TuMV-infected plants. Still, the abundance of these channels at the protein level, as well as their functionality, remains to be studied. Importantly, the authors state that the plant viruses can somewhat slow down the aphid-triggered elevations of Ca^2+^ in infected leaves (Then *et al.*, 2021). Mechanical wounding triggers a massive influx of Ca^2+^ into phloem cells, which leads to the activation of plant defense by protein plugging and callose sealing of the sieve elements. However, the gel saliva of aphids is able to limit this Ca^2+^ influx by sealing the site of penetration, which additionally reduces the loss of turgor pressure and prevents the activation of the mechanosensitive Ca^2+^ channels on the PM and subsequent occlusion of sieve elements, therefore attenuating the host plant’s defense and improving aphid feeding efficiency (van Bel *et al.*, 2014). The phloem is a major route for viral infection to spread systemically throughout the plant (Vuorinen *et al.*, 2011). Since clogging of the phloem would also restrict the long-distance transport of viruses, it can be assumed that viruses could indeed contribute to suppression of the Ca^2+^ influx caused by insect feeding. On the other hand, the rice gall dwarf virus reduces the secretion of its insect vector’s specific Ca^2+^-binding saliva protein, which leads to increased Ca^2+^ influx and subsequent callose deposition in the phloem. The insect vector encounters a stronger barrier to feeding and changes its behaviour, probing more frequently and secreting more saliva into rice plants, thus ultimately enhancing transmission of the virus between plants (Wu *et al.*, 2022). Based on these data, Ca^2+^ signaling likely plays a significant role in the horizontal transmission of these viruses. However, further research is required to provide more clarity about the regulatory mechanisms.

### Internal Ca^2+^-sensor proteins

The regulation of plant immune responses, similar to those in animals, depends highly on the activation of intracellular Ca^2+^ receptors and Ca^2+^-dependent changes in protein–protein interactions. The protein families of CPKs and CaMs/calmodulin-like proteins (CMLs), as well as calcineurin B-like proteins (CBLs) together with CBL/CBL-interacting protein kinases (CIPKs), control plant defense gene expression by altering the activity of their target proteins, including transcription factors (Yuan *et al.*, 2021). Therefore, manipulating the Ca^2+^ concentration in the cytoplasm and targeting intracellular Ca^2+^ sensors were shown to be the common strategies of host immunity suppression used by plant viruses. It has been reported that tobacco mosaic virus (TMV) uses several strategies to manipulate cytoplasmic Ca^2+^ signaling and the function of intracellular Ca^2+^ sensors during infection. Initially, TMV causes an increase of the cytosolic and nuclear Ca^2+^ concentrations in the systemic tissue of root tips, which favors plant defense. Here, the authors suggest an interesting model that elevated cytosolic Ca^2+^ concentration could trigger increased ROS production, which in turn would increase the Ca^2+^ concentration in the nucleus, presumably triggering programmed cell death and therefore restricting viral spread (Li *et al.*, 2018). It is important to elucidate the signal triggering such an effect in the systemic tissues and to know whether such a defense mechanism occurs in the primary infected tissue as well. Another study demonstrated a counter-defense mechanism of TMV by regulating the abundance of CML30 in *Nicotiana benthamiana*. TMV up-regulates the expression of the host coat protein (CP)-interacting protein L (IP-L), which binds to CML30 in a Ca^2+^-dependent manner. Such an interaction would lead to CML30 degradation and, at the same time, down-regulate its own expression. Presumably, the down-regulation of CML30 would reduce Ca^2+^-activated oxidative stress, providing a replication advantage for the virus (Liu *et al.*, 2022).

There is some indirect evidence suggesting the importance of Ca^2+^ signaling in the antiviral response, but further research is needed to establish the exact interconnection and mechanisms involved. For example, the transgenic expression of soybean CaMs in tobacco plants led to enhanced resistance against TMV, with plants demonstrating fewer and smaller HR lesions that also appeared earlier (Heo *et al.*, 1999). Interestingly, the TMV movement protein (MP) binds the Ca^2+^-binding ER chaperone calreticulin to accomplish cell-to-cell movement of the virus, and overexpression of calreticulin hinders normal interaction with MP and delays cell-to-cell movement (Fig. 1) (Chen *et al.*, 2005). There is one more piece of evidence that the Ca^2+^ concentration in the cytoplasm is a regulator of plant defense against TMV. It has been shown that polysaccharides may act as elicitors of the plant immune system by triggering Ca^2+^ influx into the cytoplasm, which presumably regulates calreticulin activity as well as ROS and SA production (Menard *et al.*, 2004; Yin *et al.*, 2010; Zhao *et al.*, 2018).

One important player in antiviral defense is the regulator of gene silencing-calmodulin-like protein (rgs-CaM), which acts as an activator of plant defense and is therefore often targeted by viruses. First, rgs-CaM was found to be involved in the development of both local and systemic acquired resistance against CMV in tobacco plants (Jeon *et al.*, 2017). It binds to a viral RNA silencing suppressor and directs it to be degraded via autophagy. The authors also stated that the interaction of rgs-CaM with the viral suppressor of RNA silencing occurs concurrently with the activation of Ca^2+^ influx into the cytoplasm. This induces defense reactions such as SA signaling, ROS generation, and cell death. Thus, rgs-CaM acts as an immune receptor. Surprisingly, in the case of tobacco etch virus (TEV) infection, rgs-CaM performs a fundamentally different function: it interacts with the viral RNA silencing suppressor protein HC-Pro and itself acts as a silencing suppressor (Anandalakshmi *et al.*, 2000). Nevertheless, the direct demonstration of a connection to Ca^2+^ signaling for this case has not been established, and it remains a matter of speculation whether HC-Pro stimulates an endogenous mechanism of Ca^2+^ regulation to suppress RNA silencing efficiently. A similar mode of RNA silencing suppression by rgs-CaM has been shown for tomato yellow leaf curl China virus (TYLCCNV) (Li *et al*., 2014). The authors demonstrated that the viral RNA silencing suppressor βC1 up-regulates the expression of rgs-CaM in *N. benthamiana*. This leads to the suppression of secondary siRNAs production, likely through down-regulation of the expression of RNA-dependent RNA polymerase 6. However, the mechanism of rgs-CaM up-regulation by βC1, and whether Ca^2+^ signaling is affected, remains unclear.

Another remarkable mechanism of CaM-dependent activation of antiviral RNA silencing and its suppression by a viral protein has been described by Wang *et al.* (2021). Insect-mediated leaf wounding triggers the influx of Ca^2+^ into the cell and activates the interaction of the CaM CAM3 with the calmodulin-binding transcription activator 3 (CAMTA3) (Fig. 1). Upon binding, CAMTA3 activates the transcription of RNA interference (RNAi) machinery components, therefore alleviating the viral infection. The protein V2 from cotton leaf curl Multan virus (CLCuMuV) is able to disrupt the CaM–CAMTA3 interaction, providing an advantage for the virus (Wang *et al.*, 2021). All these examples support the foundation of a whole new perspective for the study of Ca^2+^-dependent regulation of RNAi.

Interestingly, some changes in transcriptional regulation happen due to the activation of transcription factors that are themselves Ca^2+^ sensitive. In animals, such a regulation may not only activate the expression of defense genes, but also have a counter-defense effect, promoting viral replication or establishing persistent infection (Zhou *et al.*, 2009; Qu *et al.*, 2022). The existence of such a type of transcriptional regulation has to be considered in plants as well.

### Binding Ca^2+^ ions

Importantly, both animal (Zhou *et al.*, 2009) and plant viruses use the strategy of binding Ca^2+^ ions in order to stabilize their capsids. Such data are available for the turnip crinkle virus (TCV) (Laakso and Heaton, 1993; Lin and Heaton, 1999), TMV (Pattanayek *et al.*, 1992), tomato bushy stunt virus (Llauro *et al.*, 2015), and sesbania mosaic virus (Gopinath *et al.*, 1994). Mutation of the Ca^2+^-binding sites of TCV can affect cell-to-cell or long-distance movement and induce delayed mild systemic symptoms (Laakso and Heaton, 1993; Lin and Heaton, 1999). In animals, Ca^2+^ binding is also required for the optimal enzymatic activity and stability of some viral protein oligomers (Zhou *et al.*, 2009). Whether plant viral proteins require Ca^2+^ binding for their activity remains unclear.

## Organelle-specific regulation of Ca^2+^ signaling during viral infections

### ER, mitochondria, and chloroplasts

Another mechanism leading to attenuation of the host immune response in animals is caused by the modification of protein-trafficking pathways due to decreased Ca^2+^ concentrations in the ER and Golgi complex. Interestingly, Ca^2+^ released from the ER can be taken up by mitochondria, resulting in the so-called ER–mitochondria Ca^2+^ flux (Fig. 1). In the mitochondria, Ca^2+^ can regulate ATP synthesis, to meet the increased energy demand in the infected cell (Zhou *et al.*, 2009). However, the resulting excess of Ca^2+^ in the mitochondrial matrix causes the loss of mitochondrial potential and the release of cytochrome *c*, which in turn activates caspase 9 and leads to apoptosis. At the early and middle stages of infection, apoptosis serves as a host defense mechanism to decrease the number of infected cells and therefore restrict the infection. In order to counteract this type of defense and thereby promote viral replication, viruses employ proteins that suppress the ER–mitochondrial Ca^2+^ fluxes and, therefore, apoptosis. On the other hand, at the late stages of infection, apoptosis might be beneficial, to facilitate the release of the virions from infected cells. It has been shown that some viral proteins are able to alter the mitochondrial membrane potential. As a result, the Ca^2+^ concentration increases in the mitochondrial matrix, triggering apoptosis that might benefit the virus (Zhou *et al.*, 2009; Qu *et al.*, 2022).

We assume that in plants a similar mechanism might also involve the chloroplasts, which are one of the major Ca^2+^ depots in plant cells. Chloroplasts play an important role in the induction of cell death. It has been suggested that ROS production and the release of cytochrome *f* from chloroplasts may lead to apoptosis as a protective mechanism that limits viral spread (Van Aken and Van Breusegem, 2015). Chloroplasts are often targeted by viruses to promote viral reproduction. Accordingly, viral infections often affect chlorophyll fluorescence, photosystem efficiency, and photoassimilate accumulation; they alter the chloroplast ultrastructure as well as the expression of nuclear-encoded photosynthetic genes (Bhattacharyya and Chakraborty, 2018). Furthermore, direct binding of viral components to chloroplast proteins has been shown (Zhao *et al.*, 2016). Moreover, chloroplasts provide the required energy for defense responses and are the source of ROS, SA and jasmonic acid, and defense compounds such as phenylpropanoids (Serrano *et al.*, 2016; Bittner *et al.*, 2022).

It has been reported that the C4 protein of tomato yellow leaf curl virus (TYLCV) is relocated from the PM to the chloroplast in the course of viral infection. There, C4 interacts with the chloroplast Ca^2+^ sensor (CAS) protein, suppressing CAS-dependent immune defense, including SA biosynthesis and the cytoplasmic flg22-triggered Ca^2+^ burst (Fig. 1). Interestingly, the Ca^2+^-dependent protein kinase CPK16 is relocated in a similar manner in the plant from the PM to the chloroplast to promote a chloroplast-mediated defense response (Medina-Puche *et al.*, 2020). The localization of CPK16 to the PM is dependent on its N-terminal acylation, and suppressing only the N-terminal myristoylation was shown to be sufficient to lead to localization of CPK16 to the chloroplast (Stael *et al.*, 2011). Thus, the mechanisms of correct subcellular targeting also present effective targets for plant viruses, as these would also impair the effective function of host proteins that regulate Ca^2+^-dependent immune responses in the chloroplast. Therefore, suppressing defense processes in chloroplasts might be a key target for viruses in order to establish a successful infection.

### Plasmodesmata

An important aspect in plant immunity to viruses is the restriction of the size-exclusion limit of the plasmodesmata (PD). To facilitate cell-to-cell movement, viruses often aim to increase the size of the PD, for instance, by decreasing the amount of callose deposition in PD. This process is regulated by the activity of the PM nanodomain-associated proteins, the remorins. There is evidence that the recognition of potato virus X (PVX) CP and triple gene block 1 (TGB1) proteins trigger changes in the distribution of the remorin REM1.3 within the PM. This will increase the association of REM1.3 with PD, which leads to restriction of the movement of the virus from cell to cell (Perraki *et al.*, 2018). Previously, a partial membrane localization, dependent on N-terminal myristoylation, was shown for the Ca^2+^-dependent protein kinase CPK3 and, among others, remorin was suggested as a potential target of CPK3 (Mehlmer *et al.*, 2010). Perraki *et al.* (2018) demonstrated that REM1.3 is directly phosphorylated by CPK3 and proposed that this will increase its association with PD, which leads to restriction of the cell-to-cell movement of viruses. Similarly, the same researchers show further that the infection of Arabidopsis with plantago asiatica mosaic virus induced CPK3-dependent increase of REM1.2 diffusion in the PM, which affected the spread of infection (Jolivet *et al.*, 2023). Notably, the accumulation of AtCPK3 transcripts is significantly increased in response to various viral infections (Valmonte-Cortes *et al.*, 2022), further suggesting an important role of CPK3 in antiviral response regulation. Remorin-related regulation of cell-to-cell transport has also been shown for TuMV. The viral P3N-PIPO protein recruits the PM-associated Ca^2+^-binding host protein plasma membrane-associated cation-binding protein 1 (pCaP1) to PD. There, pCaP1 is able to depolymerize actin filaments, thereby increasing the size of PD and facilitating viral cell-to-cell movement. The remorin REM1.2 competes with pCaP1 for binding actin filaments and, therefore, restricting cell-to-cell movement. To counteract this host defense mechanism and to establish systemic infection, another TuMV protein, VPg, binds to remorin, which leads to proteasome-mediated degradation of remorin, giving an advantage to the virus (Cheng *et al.*, 2020). Nonetheless, the definitive association between remorin-dependent regulation of PD functions and Ca^2+^ signaling remains not fully confirmed in the described studies and requires further investigation.

## Outlook

In animal cells, viral proteins are able to modulate Ca^2+^ signaling to modify energy turnover, the transcriptional regulation of defense genes, and vesicle trafficking in order to promote viral replication. Furthermore, these viruses can establish latent infections to evade host defenses, to direct the infected cell into pro-survival pathways in order to inhibit the host innate immune response or, at a later stage of infection, into apoptosis to facilitate the release of new virions and further spread the infection. Interestingly, often one viral protein can affect different stages of Ca^2+^ regulation, steering it in a pro- or counter-defense direction. At the same time, the role of Ca^2+^ signaling during the plant–virus interaction is an emerging topic, which might become useful for developing new plant protection strategies. In this review, we summarized that, similar to animal viruses, plant viruses make use of their proteins to target important components of Ca^2+^ signaling in order to counteract plant defenses. As follows from Table 1, often the viral RNAi suppressor proteins have to interact with the Ca^2+^-regulated host proteins. Presumably, the hijacking of host Ca^2+^ signaling by the viral proteins has great importance for establishing efficient suppression of the major antiviral mechanism, RNAi. The detailed mechanisms for this process still have to be studied in more detail, but work carried out to date has already opened a new horizon for investigating the role of Ca^2+^signaling in developing other types of antiviral immune responses.

**Table 1.** Regulation of the Ca^2+^-dependent defense response by plant viruses

Virus	Viral Protein	Host interacting/affected protein	Effect on host immunity		Reference
			Activation	Suppression	
TMV	CP	CML30 via CP-IP-L		CML30 down-regulation Suppression of oxidative stress	Liu *et al.* (2022)
	MP	Calreticulin		Facilitation of cell-to-cell movement	Chen *et al.* (2005)
CMV	2b	rgs-CaM	Degradation of 2b by autophagy		Jeon *et al.* (2017)
TEV	HC-Pro			Suppression of RNAi	Anandalakshmi *et al.* (2000)
TYLCCNV	βC1			Suppression of RNAi	(Li *et al.*, 2014)
CLCuMuV TYLCCNV	V2	CaM–CAMTA3		Disruption of CaM–CAMTA3 interaction, suppression of RNAi	Wang *et al.* (2021)
TYLCV	C4	CAS		Suppression of SA biosynthesis and flg22-triggered Ca^2+^ burst	Medina-Puche *et al.* (2020)
PVX	CP, TGB1	CPK3 Remorin 1.3	Restriction of cell-to-cell movement		Perraki *et al.* (2018)
TuMV	P3N-PIPO	pCaP1		Facilitation of cell-to-cell movement	Cheng *et al.* (2020)
	VPg	Remorin 1.2 Binding to pCaP1 restricts cell-to-cell movement		Proteasome-mediated degradation of remorin	

It will be important to show whether any of the viral proteins directly bind to Ca^2+^ channels or pumps, as well as to find new mechanisms that regulate the activity of the intracellular Ca^2+^ sensors and therefore their downstream targets, for example, a potential phosphorylation of Ca^2+^ channels. Most of the Ca^2+^ channels that regulate plant immunity against bacteria and fungi are located on the PM and play a role in Ca^2+^ release from the apoplast (Xu *et al.*, 2022). However, unlike bacteria and fungi, and unlike animal viruses, most plant viruses are directly injected into the cell cytoplasm by their insect vectors. Therefore, it is more likely that they would need to interact with the intracellular Ca^2+^ sensors, as well as to regulate Ca^2+^ signaling inside the cell and across the organelles. Aside from the apoplast, plants have two other main Ca^2+^ storage pools: the central vacuole and, to a lesser extent, the chloroplasts (Stael *et al.*, 2012). Special attention must be paid to investigating whether Ca^2+^ signaling across these storage pools might contribute to antiviral defense (Xu *et al.*, 2022). Another important aspect to be studied is the existence of Ca^2+^-mediated signaling between organelles, similar to the ER–mitochondria Ca^2+^ flux in animal cells. The advance in genetically encoded calcium indicators (GECIs) and the development of high-resolution imaging systems will allow us to obtain more important information concerning the functions of Ca^2+^ signaling in antiviral immunity (Resentini *et al.*, 2021).

Thus, in all eukaryotes Ca^2+^ signaling regulates both the activation and the suppression of immune responses to various pathogens. We believe that revealing the novel mechanisms of such regulation might become valuable for the development of future antiviral strategies in crops. For instance, the use of chemicals that specifically suppress certain Ca^2+^channels might be considered as a potential antiviral treatment in plants.

## Abbreviations

ACA: autoinhibited Ca^2+^-ATPase
BICAT 1, 2: bivalent cation transporter 1 and 2
CAMTA3: calmodulin-binding transcription activator-3
CaM: calmodulin
CAS: chloroplast Ca^2+^ sensor protein
CAX: Ca^2+^ ATPases and Ca^2+^/H^+^ exchanger
CBL: calcineurin B-like protein
CLCuMuV: cotton leaf curl Multan virus
cMCU: chloroplast-localized mitochondrial Ca^2+^ uniporter
CML: calmodulin-like protein
CMV: cucumber mosaic virus
CNGC: cyclic nucleotide-gated channel
CPK: calcium-dependent protein kinase
ECA: ER-type Ca^2+-^ATPase
GLR: glutamate receptor-like Ca^2+^ channel
HCX: H^+^/Ca^2+^ exchanger
IP-L: CP-interacting protein L
IP3R: inositol-1,4,5-triphosphate receptor
MCA: Mid1-complementing activity channel
MCU: mitochondrial Ca^2+^ uniporter
MP: movement protein
MSL: mechanosensitive-like channel
NCX: Na^+^/Ca^2+^ exchanger
NLR: nucleotide-binding leucine-rich repeat receptor
OSCA: reduced hyperosmolarity-induced cytosolic [Ca^2+^] increase channel
pCaP1: plasma membrane-associated cation-binding protein 1
PEC 1/2: POLLUX plastid envelope ion channel 1 and 2
PM: plasma membrane
PMCA: plasma membrane Ca^2+^-ATPase
PVX: potato virus X
rgs-CaM: regulator of gene silencing-calmodulin-like protein
RNAi: RNA interference
ROS: reactive oxygen species
RyR: ryanodine receptor
SA: salicylic acid
SERCA: sarcoplasmic/endoplasmic reticulum Ca^2+^-ATPase
TCV: turnip crinkle virus
TEV: tobacco etch virus
TGB1: triple gene block 1 protein
TMV: tobacco mosaic virus
TPC: two-pore channel
TuMV: turnip mosaic virus
TYLCCNV: tomato yellow leaf curl China virus
TYLCV: tomato yellow leaf curl virus
VDAC: voltage-dependent anion channel
VGCC: voltage-gated calcium channel.

## References

[CIT0001] Aguilar E , Lozano-Duran R. 2022. Plant viruses as probes to engineer tolerance to abiotic stress in crops. Stress Biology 2, 20. 37676519 10.1007/s44154-022-00043-4 PMC10441908

[CIT0002] Aldon D , Mbengue M, Mazars C, Galaud JP. 2018. Calcium signalling in plant biotic interactions. International Journal of Molecular Sciences 19, 665. 29495448 10.3390/ijms19030665 PMC5877526

[CIT0003] Amari K , Huang C, Heinlein M. 2021. Potential impact of global warming on virus propagation in infected plants and agricultural productivity. Frontiers in Plant Science 12, 649768. 33868349 10.3389/fpls.2021.649768 PMC8045756

[CIT0004] Anandalakshmi R , Marathe R, Ge X, Herr JM Jr, Mau C, Mallory A, Pruss G, Bowman L, Vance VB. 2000. A calmodulin-related protein that suppresses posttranscriptional gene silencing in plants. Science 290, 142–144. 11021800 10.1126/science.290.5489.142

[CIT0005] Aregbesola OZ , Legg JP, Sigsgaard L, Lund OS, Rapisarda C. 2019. Potential impact of climate change on whiteflies and implications for the spread of vectored viruses. Journal of Pest Science 92, 1309–1311.

[CIT0006] Bastas KK. 2022. Impact of climate change on food security and plant disease. In: Kumar A, ed. Microbial biocontrol: food security and post harvest management. Vol. 2. Cham: Springer International Publishing, 1–22.

[CIT0007] Bhattacharyya D , Chakraborty S. 2018. Chloroplast: the Trojan horse in plant–virus interaction. Molecular Plant Pathology 19, 504–518. 28056496 10.1111/mpp.12533 PMC6638057

[CIT0008] Bittner A , Ciesla A, Gruden K, Lukan T, Mahmud S, Teige M, Vothknecht UC, Wurzinger B. 2022. Organelles and phytohormones: a network of interactions in plant stress responses. Journal of Experimental Botany 73, 7165–7181. 36169618 10.1093/jxb/erac384 PMC9675595

[CIT0009] Bjornson M , Pimprikar P, Nurnberger T, Zipfel C. 2021. The transcriptional landscape of *Arabidopsis thaliana* pattern-triggered immunity. Nature Plants 7, 579–586. 33723429 10.1038/s41477-021-00874-5 PMC7610817

[CIT0010] Calil IP , Fontes EPB. 2017. Plant immunity against viruses: antiviral immune receptors in focus. Annals of Botany 119, 711–723. 27780814 10.1093/aob/mcw200 PMC5604577

[CIT0011] Chauhan P , Singla K, Rajbhar M, Singh A, Das N, Kumar K. 2019. A systematic review of conventional and advanced approaches for the control of plant viruses. Journal of Applied Biology & Biotechnology 7, 89–98.

[CIT0012] Chen MH , Tian GW, Gafni Y, Citovsky V. 2005. Effects of calreticulin on viral cell-to-cell movement. Plant Physiology 138, 1866–1876. 16006596 10.1104/pp.105.064386 PMC1183378

[CIT0013] Cheng G , Yang Z, Zhang H, Zhang J, Xu J. 2020. Remorin interacting with PCaP1 impairs *Turnip mosaic virus* intercellular movement but is antagonised by VPg. New Phytologist 225, 2122–2139. 31657467 10.1111/nph.16285

[CIT0014] Chiu YH , Hung YL, Wang HP, Wei WL, Shang QW, Pham TH, Huang CK, Pan ZJ, Lin SS. 2021. Investigation of P1/HC-Pro-mediated ABA/calcium signaling responses via gene silencing through high- and low-throughput RNA-seq approaches. Viruses 13, 2349. 34960618 10.3390/v13122349 PMC8708664

[CIT0015] Choi WG , Miller G, Wallace I, Harper J, Mittler R, Gilroy S. 2017. Orchestrating rapid long-distance signaling in plants with Ca^2+^, ROS and electrical signals. The Plant Journal 90, 698–707. 28112437 10.1111/tpj.13492 PMC5677518

[CIT0016] Edel KH , Marchadier E, Brownlee C, Kudla J, Hetherington AM. 2017. The evolution of calcium-based signalling in plants. Current Biology 27, R667–R679. 28697370 10.1016/j.cub.2017.05.020

[CIT0017] Forderer A , Kourelis J. 2023. NLR immune receptors: structure and function in plant disease resistance. Biochemical Society Transactions 51, 1473–1483. 37602488 10.1042/BST20221087 PMC10586772

[CIT0018] Frank J , Happeck R, Meier B, Hoang MTT, Stribny J, Hause G, Ding H, Morsomme P, Baginsky S, Peiter E. 2019. Chloroplast-localized BICAT proteins shape stromal calcium signals and are required for efficient photosynthesis. New Phytologist 221, 866–880. 30169890 10.1111/nph.15407

[CIT0019] Garcia-Brugger A , Lamotte O, Vandelle E, Bourque S, Lecourieux D, Poinssot B, Wendehenne D, Pugin A. 2006. Early signaling events induced by elicitors of plant defenses. Molecular Plant-Microbe Interactions 19, 711–724. 16838784 10.1094/MPMI-19-0711

[CIT0020] Gilroy S , Bialasek M, Suzuki N, Gorecka M, Devireddy AR, Karpinski S, Mittler R. 2016. ROS, calcium, and electric signals: key mediators of rapid systemic signaling in plants. Plant Physiology 171, 1606–1615. 27208294 10.1104/pp.16.00434 PMC4936577

[CIT0021] Gopinath K , Sundareshan S, Bhuvaneswari M, Karande A, Murthy MR, Nayudu MV, Savithri HS. 1994. Primary structure of sesbania mosaic virus coat protein: its implications to the assembly and architecture of the virus. Indian Journal of Biochemistry & Biophysics 31, 322–328. 8002015

[CIT0022] Gorovits R , Shteinberg M, Anfoka G, Czosnek H. 2022. Exploiting virus infection to protect plants from abiotic stresses: tomato protection by a Begomovirus. Plants 11, 2944. 36365396 10.3390/plants11212944 PMC9657025

[CIT0023] Heo WD , Lee SH, Kim MC, et al. 1999. Involvement of specific calmodulin isoforms in salicylic acid-independent activation of plant disease resistance responses. Proceedings of the National Academy of Sciences, USA 96, 766–771. 10.1073/pnas.96.2.766 PMC15211 9892708

[CIT0024] Ivanov PA , Gasanova TV, Repina MN, Zamyatnin AA Jr. 2023. Signaling and resistosome formation in plant innate immunity to viruses: is there a common mechanism of antiviral resistance conserved across kingdoms? International Journal of Molecular Sciences 24, 13625. 37686431 10.3390/ijms241713625 PMC10487714

[CIT0025] Jeon EJ , Tadamura K, Murakami T, Inaba JI, Kim BM, Sato M, Atsumi G, Kuchitsu K, Masuta C, Nakahara KS. 2017. rgs-CaM detects and counteracts viral RNA silencing suppressors in plant immune priming. Journal of Virology 91, e00761–e00717. 28724770 10.1128/JVI.00761-17 PMC5599751

[CIT0026] Jolivet MD , Deroubaix A-F, Boudsocq M, et al. 2023. Interdependence of a kinase and its cognate substrate plasma membrane nanoscale dynamics underlies *Arabidopsis* response to viral infection. eLife 12, RP90309. 10.7554/eLife.90309 PMC12048157 40315285

[CIT0027] Jones RAC. 2021. Global plant virus disease pandemics and epidemics. Plants 10, 233. 33504044 10.3390/plants10020233 PMC7911862

[CIT0028] Koster P , DeFalco TA, Zipfel C. 2022. Ca^2+^ signals in plant immunity. The EMBO Journal 41, e110741. 35560235 10.15252/embj.2022110741 PMC9194748

[CIT0029] Laakso MM , Heaton LA. 1993. Asp → Asn substitutions in the putative calcium-binding site of the turnip crinkle virus coat protein affect virus movement in plants. Virology 197, 774–777. 8249300 10.1006/viro.1993.1655

[CIT0030] Li F , Huang C, Li Z, Zhou X. 2014. Suppression of RNA silencing by a plant DNA virus satellite requires a host calmodulin-like protein to repress *RDR6* expression. PLoS Pathogens 10, e1003921. 24516387 10.1371/journal.ppat.1003921 PMC3916407

[CIT0031] Li Y , Li Q, Hong Q, Lin Y, Mao W, Zhou S. 2018. Reactive oxygen species triggering systemic programmed cell death process via elevation of nuclear calcium ion level in tomatoes resisting tobacco mosaic virus. Plant Science 270, 166–175. 29576070 10.1016/j.plantsci.2018.02.010

[CIT0032] Lin B , Heaton LA. 1999. Mutational analyses of the putative calcium binding site and hinge of the turnip crinkle virus coat protein. Virology 259, 34–42. 10364487 10.1006/viro.1999.9742

[CIT0033] Liu C , Zhang J, Wang J, et al. 2022. Tobacco mosaic virus hijacks its coat protein-interacting protein IP-L to inhibit NbCML30, a calmodulin-like protein, to enhance its infection. The Plant Journal 112, 677–693. 36087000 10.1111/tpj.15972

[CIT0034] Llauro A , Coppari E, Imperatori F, Bizzarri AR, Caston JR, Santi L, Cannistraro S, de Pablo PJ. 2015. Calcium ions modulate the mechanics of tomato bushy stunt virus. Biophysical Journal 109, 390–397. 26200875 10.1016/j.bpj.2015.05.039 PMC4621496

[CIT0035] Luan S , Wang C. 2021. Calcium signaling mechanisms across kingdoms. Annual Review of Cell and Developmental Biology 37, 311–340. 10.1146/annurev-cellbio-120219-035210 34375534

[CIT0036] Medina-Puche L , Tan H, Dogra V, et al. 2020. A defense pathway linking plasma membrane and chloroplasts and co-opted by pathogens. Cell 182, 1109–1124.e25. 32841601 10.1016/j.cell.2020.07.020

[CIT0037] Mehlmer N , Wurzinger B, Stael S, Hofmann-Rodrigues D, Csaszar E, Pfister B, Bayer R, Teige M. 2010. The Ca^2+^-dependent protein kinase CPK3 is required for MAPK-independent salt-stress acclimation in Arabidopsis. The Plant Journal 63, 484–498. 20497378 10.1111/j.1365-313X.2010.04257.x PMC2988408

[CIT0038] Menard R , Alban S, de Ruffray P, Jamois F, Franz G, Fritig B, Yvin JC, Kauffmann S. 2004. β-1,3 glucan sulfate, but not β-1,3 glucan, induces the salicylic acid signaling pathway in tobacco and Arabidopsis. The Plant Cell 16, 3020–3032. 15494557 10.1105/tpc.104.024968 PMC527195

[CIT0039] Pattanayek R , Elrod M, Stubbs G. 1992. Characterization of a putative calcium-binding site in tobacco mosaic virus. Proteins 12, 128–132. 1603802 10.1002/prot.340120206

[CIT0040] Perraki A , Gronnier J, Gouguet P, et al. 2018. REM13’s phospho-status defines its plasma membrane nanodomain organization and activity in restricting PVX cell-to-cell movement. PLoS Pathogens 14, e1007378. 30419072 10.1371/journal.ppat.1007378 PMC6258466

[CIT0041] Pirayesh N , Giridhar M, Ben Khedher A, Vothknecht UC, Chigri F. 2021. Organellar calcium signaling in plants: an update. Biochimica et Biophysica Acta, Molecular Cell Research 1868, 118948. 33421535 10.1016/j.bbamcr.2021.118948

[CIT0042] Qu Y , Sun Y, Yang Z, Ding C. 2022. Calcium ions signaling: targets for attack and utilization by viruses. Frontiers in Microbiology 13, 889374. 35859744 10.3389/fmicb.2022.889374 PMC9289559

[CIT0043] Resentini F , Ruberti C, Grenzi M, Bonza MC, Costa A. 2021. The signatures of organellar calcium. Plant Physiology 187, 1985–2004. 33905517 10.1093/plphys/kiab189 PMC8644629

[CIT0044] Rubio L , Galipienso L, Ferriol I. 2020. Detection of plant viruses and disease management: relevance of genetic diversity and evolution. Frontiers in Plant Science 11, 1092. 32765569 10.3389/fpls.2020.01092 PMC7380168

[CIT0045] Schonknecht G. 2013. Calcium signals from the vacuole. Plants 2, 589–614. 27137394 10.3390/plants2040589 PMC4844392

[CIT0046] Serrano I , Audran C, Rivas S. 2016. Chloroplasts at work during plant innate immunity. Journal of Experimental Botany 67, 3845–3854. 26994477 10.1093/jxb/erw088

[CIT0047] Shepherd S , Yuen ELH, Carella P, Bozkurt TO. 2023. The wheels of destruction: plant NLR immune receptors are mobile and structurally dynamic disease resistance proteins. Current Opinion in Plant Biology 74, 102372. 37172365 10.1016/j.pbi.2023.102372

[CIT0048] Skendzic S , Zovko M, Zivkovic IP, Lesic V, Lemic D. 2021. The impact of climate change on agricultural insect pests. Insects 12, 440. 34066138 10.3390/insects12050440 PMC8150874

[CIT0049] Stael S , Bayer RG, Mehlmer N, Teige M. 2011. Protein N-acylation overrides differing targeting signals. FEBS Letters 585, 517–522. 21219905 10.1016/j.febslet.2011.01.001 PMC3971372

[CIT0050] Stael S , Wurzinger B, Mair A, Mehlmer N, Vothknecht UC, Teige M. 2012. Plant organellar calcium signalling: an emerging field. Journal of Experimental Botany 63, 1525–1542. 22200666 10.1093/jxb/err394 PMC3966264

[CIT0051] Then C , Bellegarde F, Schivre G, Martiniere A, Macia JL, Xiong TC, Drucker M. 2021. Plant viruses can alter aphid-triggered calcium elevations in infected leaves. Cells 10, 3534. 34944040 10.3390/cells10123534 PMC8700420

[CIT0052] Trebicki P. 2020. Climate change and plant virus epidemiology. Virus Research 286, 198059. 32561376 10.1016/j.virusres.2020.198059

[CIT0053] Valmonte-Cortes GR , Lilly ST, Pearson MN, Higgins CM, MacDiarmid RM. 2022. The potential of molecular indicators of plant virus infection: are plants able to tell us they are infected? Plants 11, 188. 35050076 10.3390/plants11020188 PMC8777591

[CIT0054] Van Aken O , Van Breusegem F. 2015. Licensed to kill: mitochondria, chloroplasts, and cell death. Trends in Plant Science 20, 754–766. 26442680 10.1016/j.tplants.2015.08.002

[CIT0055] van Bel AJ , Furch AC, Will T, Buxa SV, Musetti R, Hafke JB. 2014. Spread the news: systemic dissemination and local impact of Ca^2+^ signals along the phloem pathway. Journal of Experimental Botany 65, 1761–1787. 24482370 10.1093/jxb/ert425

[CIT0056] Volkner C , Holzner LJ, Day PM, Ashok AD, Vries J, Bolter B, Kunz HH. 2021. Two plastid POLLUX ion channel-like proteins are required for stress-triggered stromal Ca^2+^release. Plant Physiology 187, 2110–2125. 34618095 10.1093/plphys/kiab424 PMC8644588

[CIT0057] Vuorinen AL , Kelloniemi J, Valkonen JP. 2011. Why do viruses need phloem for systemic invasion of plants? Plant Science 181, 355–363. 21889041 10.1016/j.plantsci.2011.06.008

[CIT0058] Wang Y , Gong Q, Wu Y, et al. 2021. A calmodulin-binding transcription factor links calcium signaling to antiviral RNAi defense in plants. Cell Host & Microbe 29, 1393–1406.e7. 34352216 10.1016/j.chom.2021.07.003

[CIT0059] Wu X , Valli A, Garcia JA, Zhou X, Cheng X. 2019. The tug-of-war between plants and viruses: great progress and many remaining questions. Viruses 11, 203. 30823402 10.3390/v11030203 PMC6466000

[CIT0060] Wu W , Yi G, Lv X, Mao Q, Wei T. 2022. A leafhopper saliva protein mediates horizontal transmission of viral pathogens from insect vectors into rice phloem. Communications Biology 5, 204. 35246603 10.1038/s42003-022-03160-y PMC8897447

[CIT0061] Xu G , Moeder W, Yoshioka K, Shan L. 2022. A tale of many families: calcium channels in plant immunity. The Plant Cell 34, 1551–1567. 35134212 10.1093/plcell/koac033 PMC9048905

[CIT0062] Yin H , Zhou X, Du Y. 2010. Oligochitosan: a plant diseases vaccine—a review. Carbohydrate Polymers 82, 1–8.

[CIT0063] Yuan P , Tanaka K, Poovaiah BW. 2021. Calcium/calmodulin-mediated defense signaling: what is looming on the horizon for AtSR1/CAMTA3-mediated signaling in plant immunity. Frontiers in Plant Science 12, 795353. 35087556 10.3389/fpls.2021.795353 PMC8787297

[CIT0064] Zhang Z , Tong X, Liu SY, Chai LX, Zhu FF, Zhang XP, Zou JZ, Wang XB. 2019. Genetic analysis of a Piezo-like protein suppressing systemic movement of plant viruses in *Arabidopsis thaliana*. Scientific Reports 9, 3187. 30816193 10.1038/s41598-019-39436-3 PMC6395819

[CIT0065] Zhao J , Zhang X, Hong Y, Liu Y. 2016. Chloroplast in plant-virus interaction. Frontiers in Microbiology 7, 1565. 27757106 10.3389/fmicb.2016.01565 PMC5047884

[CIT0066] Zhao L , Chen Y, Yang W, Zhang Y, Chen W, Feng C, Wang Q, Wu Y. 2018. Polysaccharide peptide-induced virus resistance depends on Ca^2+^ influx by increasing the salicylic acid content and upregulating the leucine-rich repeat gene in *Arabidopsis thaliana*. Molecular Plant-Microbe Interactions 31, 516–524. 29199889 10.1094/MPMI-10-17-0242-R

[CIT0067] Zhou Y , Frey TK, Yang JJ. 2009. Viral calciomics: interplays between Ca^2+^ and virus. Cell Calcium 46, 1–17. 19535138 10.1016/j.ceca.2009.05.005 PMC3449087

